# *Stenotrophomonas oleivorans* sp. nov., a polycyclic aromatic hydrocarbon-degrading bacterium isolated from crude oil-contaminated soil in Tabasco, Mexico

**DOI:** 10.3389/fmicb.2026.1793198

**Published:** 2026-05-14

**Authors:** Temidayo O. Elufisan, Isabel C. Rodriguez-Luna, Alejandro Sanchez-Varela, Virginia P. Bustos, Luis Lozano, Edgar Dantan-Gonzalez, Omotayo O. Oyedara, Jose Correa-Basurto, Alan R. Estrada-Perez, Diana V. Cortes-Espinosa, Miguel A. Villalobos-Lopez, Xianwu Guo

**Affiliations:** 1 OAK Ridge Institute for Science and Education (ORISE), Oak Ridge, TN, United States; 2 Instituto Politécnico Nacional-Centro de Biotecnología Genómica, Reynosa, Tamaulipas, Mexico; 3 Centro de Ciencia Genomica Universidad Nacional Autonoma de Mexico (UNAM), Cuernavaca, Morelos, Mexico; 4 Universidad Autonoma de Estado Morelos, (UAEM), Cuernavaca, Mexico; 5 Department of Biotechnology, Osun State University, Oshogbo, Nigeria; 6 Escuela Superior de Medicinial Instituto Politecnico Nacional, Mexico City, Mexico; 7 Instituto Politecnico Nacional, Centro de Investigación en Biotecnología Aplicada (IPN-CIBA), Tepetitla, Tlaxcala, Mexico

**Keywords:** 16S rRNA gene, average nucleotide identity, comparative genomics, digital DNA–DNA hybridization (dDDH), novel species, polycyclic aromatic hydrocarbons (PAHs), *Stenotrophomonas oleivorans* sp. nov

## Abstract

**Introduction:**

Crude oil-contaminated soils harbor bacteria with specialized hydrocarbon-degrading capabilities, yet the *Stenotrophomonas* genus remains incompletely characterized at the species level. Strain ASS1, a motile Gram-negative rod, was isolated from crude oil-contaminated soil in Tabasco, Mexico, and initial 16S rRNA gene sequencing indicated affiliation with *Stenotrophomonas*, warranting full polyphasic characterization.

**Methods:**

The complete genome was sequenced using Illumina MiSeq and assembled into a single closed contig (4,564,481 bp; G+C content 66.59%; GenBank: CP031167). Genome quality was assessed with BUSCO v6.0.0 and CheckM2. Species boundaries were evaluated using average nucleotide identity (ANIb via JSpeciesWS) and digital DNA–DNA hybridization (dDDH via GGDC Formula 2) against 38 representative *Stenotrophomonas genomes*. Taxonomic placement was further resolved by TYGS, MLST, and core genome phylogenomics. PAH catabolic capacity was assessed through genome annotation and in vitro growth assays on four PAHs as sole carbon sources, analyzed by Kruskal–Wallis test.

**Results:**

BUSCO completeness was 99.7% and CheckM2 confirmed 100.0% completeness with 0.0% contamination. Despite 99.86% 16S rRNA gene identity to *Stenotrophomonas geniculata* ATCC 19374T, ANIb against the closest neighbor Stenotrophomonas *riyadhensis* CFS3442T was 93.30%, and dDDH was 50.4% (95% CI: 47.8–53.1%), both below established species thresholds. No pairwise comparison across the 38 genomes exceeded 54.3% dDDH. TYGS, MLST, and core genome phylogenomics consistently resolved ASS1 as a distinct lineage. Pan genome analysis confirmed an open pan genome (alpha = 0.485). A complete upper pathway PAH catabolic gene cassette was identified, and in vitro growth was statistically equivalent across all four PAH substrates (Kruskal–Wallis H(3) = 2.403, *p* = 0.493).

**Discussion:**

Collectively, the genomic, phylogenomic, and phenotypic evidence supports ASS1 as the type strain of a novel species, *Stenotrophomonas oleivorans* sp. nov. (= WFCC 1006/CM-CNRG 934T), with demonstrated broad spectrum PAH degradation capacity relevant to bioremediation.

## 1 Introduction

*Stenotrophomonas* is a genus of non-fermentative, Gram-negative bacteria in the class Gammaproteobacteria (Ryan et al., 2009). The first member of the genus was isolated as *Bacterium bookeri* by Edwards (1943) from the pleural fluid of a patient with oral carcinoma (Hugh and Ryschenkow, 1961; Denton and Kerr, 1998). *Bacterium bookeri* and five previously identified *Pseudomonas alcaligenes* strains were reclassified *as Pseudomonas maltophilia* (Hugh and Leifson, 1963). In Komagata et al. (1974) reviewed the classification of four *Pseudomonas melanogena* strains and determined that they were *P. maltophilia* based on their shared deoxyribonuclease and nucleoside phosphotransferase activities (Hugh and Leifson, 1963; Komagata et al., 1974). Similarly, certain bacteria previously classified *as Alcaligenes faecalis* were reclassified *as P. maltophilia* (Ulrich and Needham, 1953).

In 1973, DNA-rRNA hybridization analysis revealed that the genus *Pseudomonas* comprises five distinct rRNA homology groups. 16S rRNA cistron analysis of the type strain *Pseudomonas maltophilia* ATCC 13637^T^ *showed the closest phylogenetic relatedness to members of the genus Xanthomonas* (Palleroni et al., 1973). Building on this, Swings et al. (2009) proposed a transfer of *P. maltophilia to Xanthomonas based on DNA-rRNA hybridization, G + C content (P. maltophilia 63–67.5%; Xanthomonas 63–70%),* comparative enzymology (notably the absence of NADP-linked dehydrogenases), ubiquinone composition (both *P. maltophilia* and *Xanthomonas* spp. possess eight-isoprene ubiquinone versus nine isoprene units in other *Pseudomonas* strains), cellular fatty acid composition, whole-cell protein patterns, and isoelectric focusing of outer-membrane esterases (Swings et al., 2009). An extensive DNA-rRNA hybridization study yielded divergent melting points across 27 *Pseudomonas* strains, and Yang et al. (1993) demonstrated that *P. maltophilia* differs from *Xanthomonas* in both cellular fatty acid and polyamine compositions (Yang et al., 1993).

The inability to accommodate *P. maltophilia satisfactorily within Xanthomonas led* Palleroni and Bradbury (1993) to establish the new genus *Stenotrophomonas*, with *Stenotrophomonas maltophilia* as the sole founding species. The genus name reflects the initially observed narrow substrate utilization of *S. maltophilia* (Greek: stenos, narrow; troph, nutrition; monas, unit). This reclassification was corroborated by Nesme et al. (1995), who used restriction mapping of PCR-amplified 16S rRNA gene sequences to distinguish between *Xanthomonas* and *Stenotrophomonas* unambiguously (Nesme et al., 1995).

Although *Stenotrophomonas* was initially associated primarily with pathogenicity, owing to the clinical prominence of *S. maltophilia*, its isolation from diverse non-clinical environments has revealed numerous non-pathogenic species (Ryan et al., 2009; Geng et al., 2010). The genus has undergone successive taxonomic revisions, including the transfer of *Stenotrophomonas dokdonensis* to *Pseudoxanthomonas dokdonensis* and the re-evaluation of *Stenotrophomonas africana* as a later synonym of *S. maltophilia* (Coenye et al., 2004; Lee et al., 2008). Following a pan-genomic analysis of 19 sequenced genomes, Patil et al. (2018) proposed that *S. maltophilia* and related taxa, including *S. africana*, *Stenotrophomonas geniculata*, *Stenotrophomonas beteli, Stenotrophomonas pavanii*, and *Stenotrophomonas sepilia,* could be regrouped as the *S. maltophilia complex* (Smc) (Patil et al., 2018; Gautam et al., 2021a). To date, the genus contains more than 27 validly published species names and 43 child taxa (LPSN).^1^

The advent of whole-genome sequencing has transformed bacterial taxonomy. Taxogenomics employs genome-sequence-based tools, including average nucleotide identity (ANI), digital DNA–DNA hybridization (dDDH), multi-locus sequence typing (MLST), phylogenetic reconstruction, and average amino acid identity (AAI), to delineate species boundaries with greater precision than phenotypic or single-locus approaches (Oyedara et al., 2016; Patil et al., 2018; Elufisan et al., 2019, 2020b). The current genomic species thresholds are ANI > =95–96% and dDDH > = 70% (Richter and Rosselló-Móra, 2009). Pan-genome analysis of the genus *Stenotrophomonas* has confirmed an open pan-genome (alpha < 1), indicating that novel *Stenotrophomonas* species remain to be discovered (Patil et al., 2018). Some genes that code for enzymes involved in polycyclic aromatic hydrocarbon (PAH) catabolism, including naphthalene 1,2-dioxygenase, 1,2-dihydroxy-1,2-dihydronaphthalene dehydrogenase, 2-hydroxychromene-2-carboxylate isomerase, and salicylaldehyde dehydrogenase, have been identified in environmental *Stenotrophomonas* isolates, highlighting the biotechnological relevance of the genus for bioremediation applications (Mangwani et al., 2014; Elufisan et al., 2020b).

Here, we report the complete polyphasic characterization of strain ASS1^T^, isolated from crude oil-contaminated soil in Tabasco, Mexico, encompassing morphological, physiological, chemotaxonomic, phylogenetic, and comparative genomic analyses. Based on this evidence, we formally propose a novel species, *Stenotrophomonas oleivorans* sp. nov., with strain ASS1^T^ as the type strain.

## 2 Materials and methods

### 2.1 Isolation and growth conditions

Strain ASS1 was isolated from crude oil-contaminated soil at 17^o^ 52′ 26.9” N, 92^o^ 29′ 12.4” W, Tabasco, Mexico. Soil (~10 g) was suspended in 90 mL sterile water, agitated at 150 rpm for 1 h, centrifuged at 3,000 x g for 20 min, and the supernatant was serially diluted (10^−1^ to 10^−8^). Dilutions were enriched in LB broth at 30 °*C* for 24 h, plated *on Stenotrophomonas* Vancomycin Imipenem (SVIA) (Himedia, India) agar, and incubated at 30 °C. The temperature range was tested at 10–42 °C, and the pH range was at pH 4–10.

### 2.2 Morphological, physiological, and MALDI-TOF MS identification

Cell morphology was assessed using light microscopy (Olympus BX51) and scanning electron microscopy (SEM; 8% paraformaldehyde fixation, critical-point dried, and gold-coated). Motility was assessed using the hanging-drop method. Biochemical characterization was performed using the conventional methods described in Bergey’s manual (Breed Robert et al., 1957; Aladame, 1987). Antimicrobial susceptibility was determined by disc diffusion on Mueller-Hinton agar according to CLSI guidelines (Patel, 2016).

Matrix-Assisted Laser Desorption/Ionization Time-of-Flight Mass Spectrometry (MALDI-TOF MS) identification was performed using a Bruker Biotyper system (Bruker Daltonics, Germany). For direct deposition, a single colony was transferred to a polished steel target and overlaid with 1 uL matrix solution (alpha-cyano-4-hydroxycinnamic acid in 50% acetonitrile / 2.5% TFA). The formic acid extraction method was used for confirmation: colonies were resuspended in 300 uL water, mixed with 900 uL absolute ethanol, centrifuged (12,000 x g, 2 min), pellet air-dried, and resuspended in 10 uL 70% formic acid and 10 uL acetonitrile; 1 uL was spotted and overlaid with matrix. Spectra acquired at 2,000–20,000 Da; identification against the Bruker MBT Compass Library v. 8.0.

### 2.3 Chemotaxonomic characterization: fatty acid analysis

Cellular fatty acids were extracted by saponification-methylation (Brian and Gardner, 1967) and analyzed by UHPLC-ESI-QTOF-MS (Agilent 1,290 Infinity II / Q-TOF 6545A; Acquity UPLC BEH C18 column, 2.1 × 50 mm, 1.7 um; Waters) at 50 ± 0.5 °C. Gradient: 0.1% formic acid in water (A) and 0.1% formic acid in methanol (B) at 0.3 mL/min. Data were processed using the MassHunter Qualitative Analysis v. B.07.00, using the Find by Formula algorithm (Sánchez-Castro et al., 2017). Compound identification was performed using the LIPID MAPS Structure Database (LMSD; www.lipidmaps.org) and Agilent Mass Hunter METLIN library (METLIN-PCDL v. 2018) with an accurate mass tolerance of <5 ppm.

### 2.4 Genome sequencing, assembly, and quality assessment

Genomic DNA was extracted using a Promega Wizard DNA extraction kit (Promega, USA). Whole-genome sequencing was performed on the Illumina MiSeq platform (2 × 300 bp paired-end). Reads were assembled using SPAdes v. 3.12.0 (Bankevich et al., 2012). Genome completeness was evaluated using BUSCO v. 6.0.0 (Manni et al., 2021) and CheckM2 (Chklovski et al., 2023). Contamination was screened using ContEst16S (Lee et al., 2017). Annotation was performed using Prokka v. 1.14 (Seemann, 2014) and NCBI PGAP (Tatusova et al., 2016). The circular genome map was generated using Proksee (Grant et al., 2023).

### 2.5 Phylogenetic and taxogenomic analyses

The complete 16S rRNA gene (GenBank: ON323668) was retrieved via EzBioCloud ContEst16S, which determines the presence of contamination in the bacterial genomes (Lee et al., 2017). A Maximum Likelihood (ML) tree was constructed in MEGA XII using the Kimura 2-parameter (K2P) model with 1,000 bootstrap replicates; *Lysobacter enzymogenes* was used as an outgroup. MLST-based phylogeny used 82 concatenated loci via autoMLST.^2^ Core genome phylogeny using BPGA v. 1.3 (Chaudhari et al., 2016) aligned in MEGA XII (ML, K2P model, 1,000 bootstrap replicates). ANI was calculated using the ANIb algorithm in JSpeciesWS (Richter et al., 2016) against all available *Stenotrophomonas* type-strain genomes. dDDH was computed using GGDC v. 3.0 (Meier-Kolthoff et al., 2013) for ASS1^T^ against 38 representative *Stenotrophomonas* and related genomes; Formula 2 (d4) was used as the primary estimate per Meier-Kolthoff et al. (2013), with 95% confidence intervals calculated from 1,000 bootstrap replicates. Species novelty was corroborated by TYGS (Meier-Kolthoff and Göker, 2019). Species thresholds: ANI > =95–96% and dDDH > = 70% (Auch et al., 2010; Richter et al., 2016; Elbir, 2024).

### 2.6 Pan-genome analysis

Pan-genome analysis of ASS1^T^ and 24 representative *Stenotrophomonas* genomes was performed using BPGA v. 1.3 (Chaudhari et al., 2016). Core, accessory, and unique genes were defined as present in 100%, 2–99, and 1% of genomes, respectively. The openness parameter alpha was 0.485, indicating an open pan-genome. Gene functions annotated using eggNOG-mapper v. 2.1; pathways mapped on KEGG via JGI-IMG (Mavromatis et al., 2009).

### 2.7 PAH degradation genes and BGC prediction

PAH-related genes were identified using BLASTp against the NCBI non-redundant protein database and KEGG pathway mapping on JGI-IMG. BGCs were predicted using antiSMASH v. 6.0 (Blin et al., 2021).

### 2.8 PAH growth assays

ASS1^T^ growth on PAHs as the sole carbon source was evaluated in Bushnell-Haas (BH) minimal medium supplemented with anthracene, anthraquinone, biphenyl, naphthalene, phenanthridine, phenanthrene, or xylene (1 and 5 mg/mL). PAHs were dissolved in anhydrous dichloromethane (DCM; ACS grade); DCM was allowed to evaporate completely under a class II biosafety cabinet (minimum 2 h; confirmed gravimetrically within 1 mg) before the addition of sterile BH medium. Controls: uninoculated BH + PAH; BH + ASS1^T^ without a carbon source. Incubation: 30 °C at 200 rpm, 25 days; growth monitored every 2 days by colony counting (10^−4^ dilution, LB agar, 30 °C for 24 h) and OD600.

Statistics: Shapiro–Wilk normality tests per PAH dataset; Levene’s test for homogeneity of variance. Primary analysis: Kruskal–Wallis test (normality violated in three of the four datasets, *p* < 0.05) with Bonferroni-corrected Mann–Whitney U pairwise tests (corrected alpha = 0.0083). Parametric corroboration: one-way ANOVA. All analyses were performed using Python v. 3.11 (SciPy v. 1.11).

## 3 Results

### 3.1 Morphological and physiological characteristics

**Figure 1:**
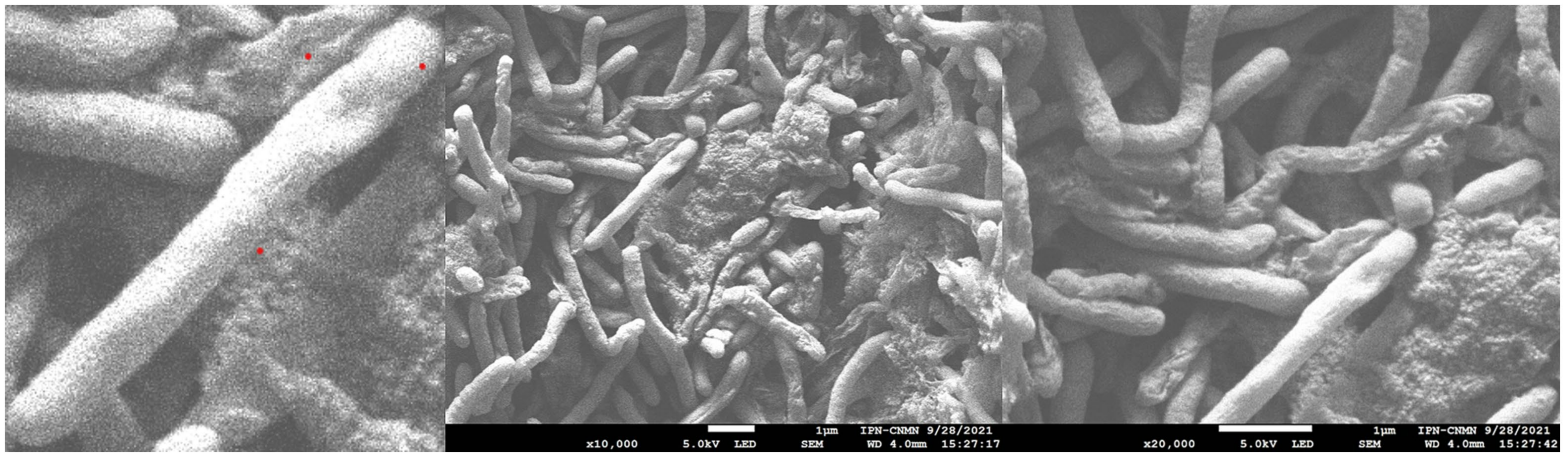
Scanning electron microscopy (SEM) images of *Stenotrophomonas oleivorans* sp. nov. ASS1T. The cells were fixed with 8% paraformaldehyde and critical-point dried. Scale bar = 1 μm (10,000× magnification, 5.0 kV).

Strain ASS1^T^ appeared as elongated rods under SEM (Figure 1). Colonies on LB and SVIA agar were yellow, slightly raised, and smooth after 48 h at 37 °*C*; colorless on MacConkey agar, confirming that the strain is a non-lactose fermenter, consistent with the genus *Stenotrophomonas.* Growth occurred at 25–37 °C (optimum 37 °C) and pH 6–8 (optimum pH 7–8). MALDI-TOF MS identification (Bruker Biotyper, MBT Compass Library, v. 8.0) was performed at an external facility. Genus-level assignment was independently confirmed by 16S rRNA sequence analysis and genome-based phylogenomics.

Further biochemical and physiological characterization of strain ASS1ᵀ revealed the following features.

ASS1^T^ is Gram-negative, catalase-positive, and oxidase-negative. It hydrolyzes starch, Tween 80, and aesculin, but not gelatin, distinguishing it from *S. geniculata* ATCC 19374^T^ and *S. hibiscicola* ATCC 19867^T^, both of which hydrolyze gelatin. ASS1^T^ utilizes arabinose, glucose, fructose, mannose, galactose, lysine, and citrate as carbon sources; galactose utilization is absent in both reference strains. Complete biochemical profile: Table 1. Antimicrobial susceptibility: Resistance to sulfamethoxazole-trimethoprim, chloramphenicol, imipenem, ceftriaxone, ceftazidime, ampicillin, doxycycline, gentamicin, and nitrofurantoin, and susceptibility to levofloxacin and ofloxacin (Elufisan et al., 2020a).

**Table 1:** Phenotypic and biochemical characteristics of *Stenotrophomonas* sp. ASS1.

Biochemical tests	1	2	3	4	5	6	7	8	9	10	11	12	13	14
Oxidase	−	−	−	−	+	+	+	+	−	−	+	−	−	−
Growth at														
4^o^ C	−		−	−	−	−	−	−	−	ND	+	−	−	−
40 ^o^ C	−	−	−	−	−	−	−	+	−					
pH 6–8	+	ND	ND	ND	ND	ND	ND	ND	ND	ND	ND	ND	ND	ND
Assimilation of														
L-Arabinose	+	ND	+	−	+	−	−	−	−	ND	+	−	W	W
Dulcitol	−	ND	ND	ND	ND	ND	ND	ND	ND	ND	ND	ND	ND	ND
Galactose	+	ND	+	−	−	−	−	−	−	−	−	+	−	−
Glucose	+	+	+	−	+	−	+	−	+	+	+	−	+	+
Lactose	−	−	ND	−	−	-	−	−	−	+	−	−	−	−
Maltose	+	+	+	−	−	+	−	−	+	+	+	+	+	+
Fructose	+	+	ND	−	+	+	+							
Mannitol	+	−	+	ND	ND	ND	ND	ND	ND	ND	ND	ND	ND	ND
Mannose	+	+	−	+	+	−	+	−	+	+	+	−	+	+
Sucrose	−	+	−	−	−		−	−	+	+	−	−	−	−
Trehalose	+	−	−	ND	ND	ND	ND	ND	ND	ND	ND	ND	ND	ND
Citrate	+	+	+	+	+	+	+							
lysine	+	ND	ND	ND	ND	ND	ND	ND	ND	ND	ND	ND	ND	ND
Phenyalalanine	–	ND	ND	ND	ND	ND	ND	ND	ND	ND	ND	ND	ND	ND
Hydrolysis of														
Starch	+	−		ND	ND	ND	ND	ND	ND	ND	ND	ND	ND	ND
Tween 80	+	+	+	+	+	−	−	+	+	−	+	ND	ND	ND
Aescuelin	+	ND	+	+	−	−	−	−	w	+	+	+	+	+
Gelatin	−	+	+	+	+	w	−	+	+	+	+	+	+	+

Data for strains 3, 13, 14, and 15 were obtained from Gautam et al. (2021b), whereas data 4–12 were obtained from Ramos et al. (2011).

+ can utilize sugar or amino acid for growth, w = weak utilization of substrate.

− cannot use amino acid or carbon source for growth, ND, not determined.

1. *S. oleivoranas* ASS1 2. *S. sepilia* strain SM16975, 3. *S. pavanii* sp. nov. ICB 89 T; 4. *S. terrae* LMG 23958 T; 5. *S. humi* LMG 23959 T; 6. *S. nitritireducens* LMG 22074 T; 7. *S. acidaminiphila* LMG 22073 T; 8. *S. koreensis* LMG 23369 T; 9. *S. maltophilia* LMG 958 T; 10. *S. maltophilia* LMG 22072; 11. *S. rhizophila* LMG 22075 T; 12. *P. beteli* LMG 00978 T; 13. *P. hibiscicola* JCM13361T; 14. *P. geniculata* JCM13324T.

### 3.2 Fatty acid profile

LC-ESI-QTOF-MS analysis revealed that the fatty acid profile was dominated by C16:0, iso-C12:0, iso-C16:0, iso-C17:1w9c, C18:0, C18:1w9c, and C19 (Supplementary Table S1). The complete quantitative fatty acid data, including comparative profiles for *S. geniculata* ATCC 19374^T^ and *P. hibiscicola* ATCC 19867^T^, are provided in Supplementary Table S1. ASS1^T^ lacks C10:0, iso-C11:0, iso-C11:0 3-OH, iso-C13:0 3-OH, C13:0 2-OH, anteiso-C15:0, and C15:0 fatty acids present in both reference strains. Conversely, iso-C16:0, iso-C17:1w9c, C18:1w9c, and C19 are present in ASS1^T^ but absent from both reference strains, further distinguishing ASS1^T^ at the species level (Řezanka et al., 2012; Sánchez-Castro et al., 2017).

### 3.3 Genome features and quality

**Figure 2:**
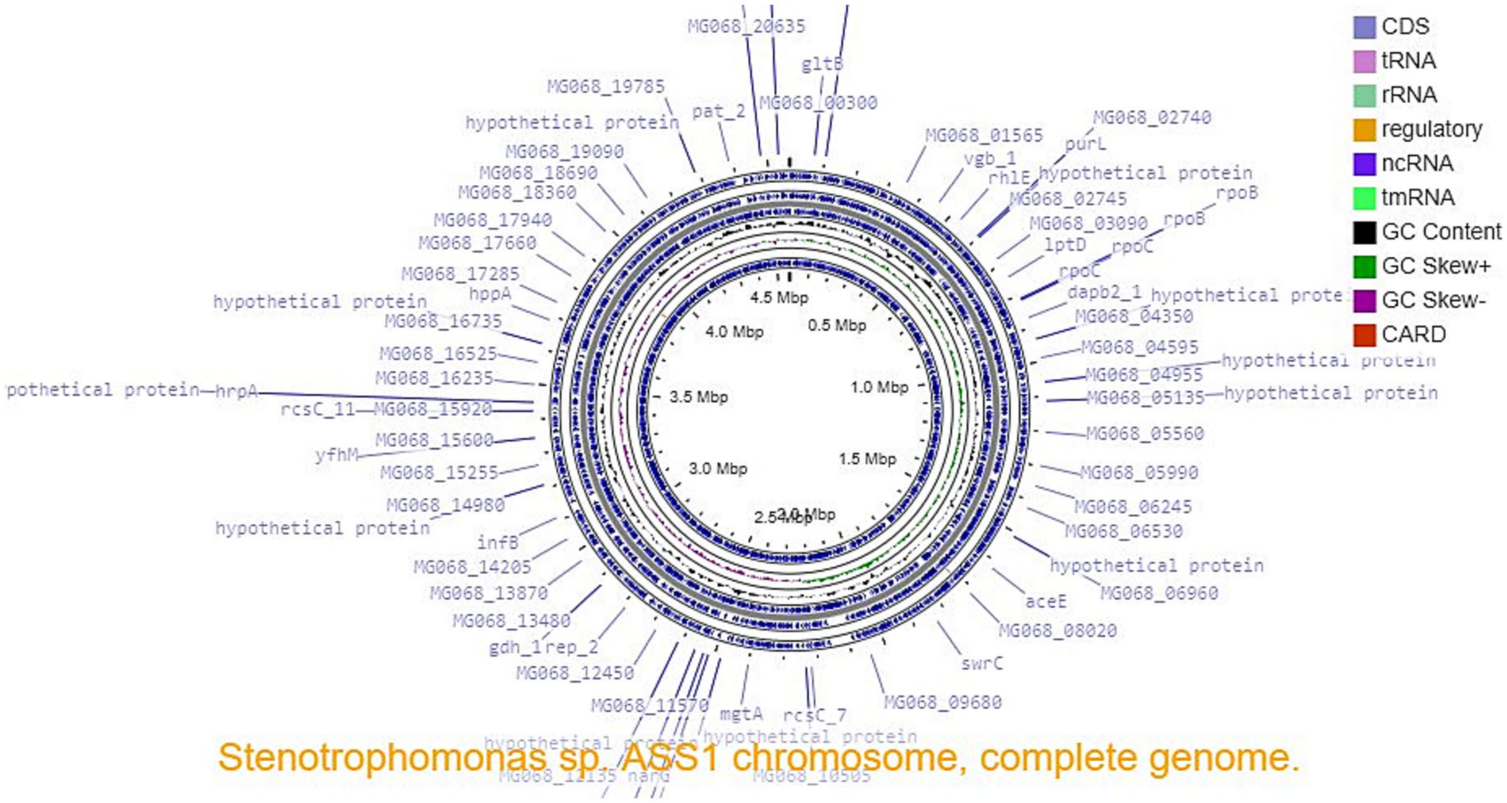
Circular genome map of *Stenotrophomonas oleivorans* sp. nov. ASS1T (GenBank: CP031167; 4,564,481 bp), which was generated using Proksee. External rings: (1) CDS forward strand; (2) CDS reverse strand; (3) tRNA (green); (4) rRNA (red); (5) regulatory elements; (6) GC content; (7) GC skew; and (8) CARD resistance genes.

The complete genome of ASS1^T^ (GenBank: CP031167) consists of 4,564,481 bp assembled into a single contig (N50 = 4,564,481 bp; total contigs = 1; maximum contig length = 4,564,481 bp), confirming a closed, complete chromosome. G + C content: 66.59%. BUSCO v. 6.0.0 (gammaproteobacteria_odb10; n = 366): completeness 99.7% [single-copy 98.9%, duplicated 0.8%, fragmented 0.0%, missing 0.3% (1 BUSCO)]. CheckM2 (neural network-specific model, translation Table 11): completeness of 100.0%, contamination of 0.0%, confirming an uncontaminated closed genome suitable for taxogenomic analysis. Prokka annotation: 4,104 CDS (coding density 0.893; average gene length 331.4 aa), 69 tRNA genes, and 9 rRNA operons. No contamination was detected using ContEst16S. Six BGCs predicted using antiSMASH v. 6.0: arylpolyene, two bacteriocins, lantipeptide, lassopeptide, and NRPS clusters. A circular genome map is shown in Figure 2.

### 3.4 Phylogenetic analyses

**Figure 3:**
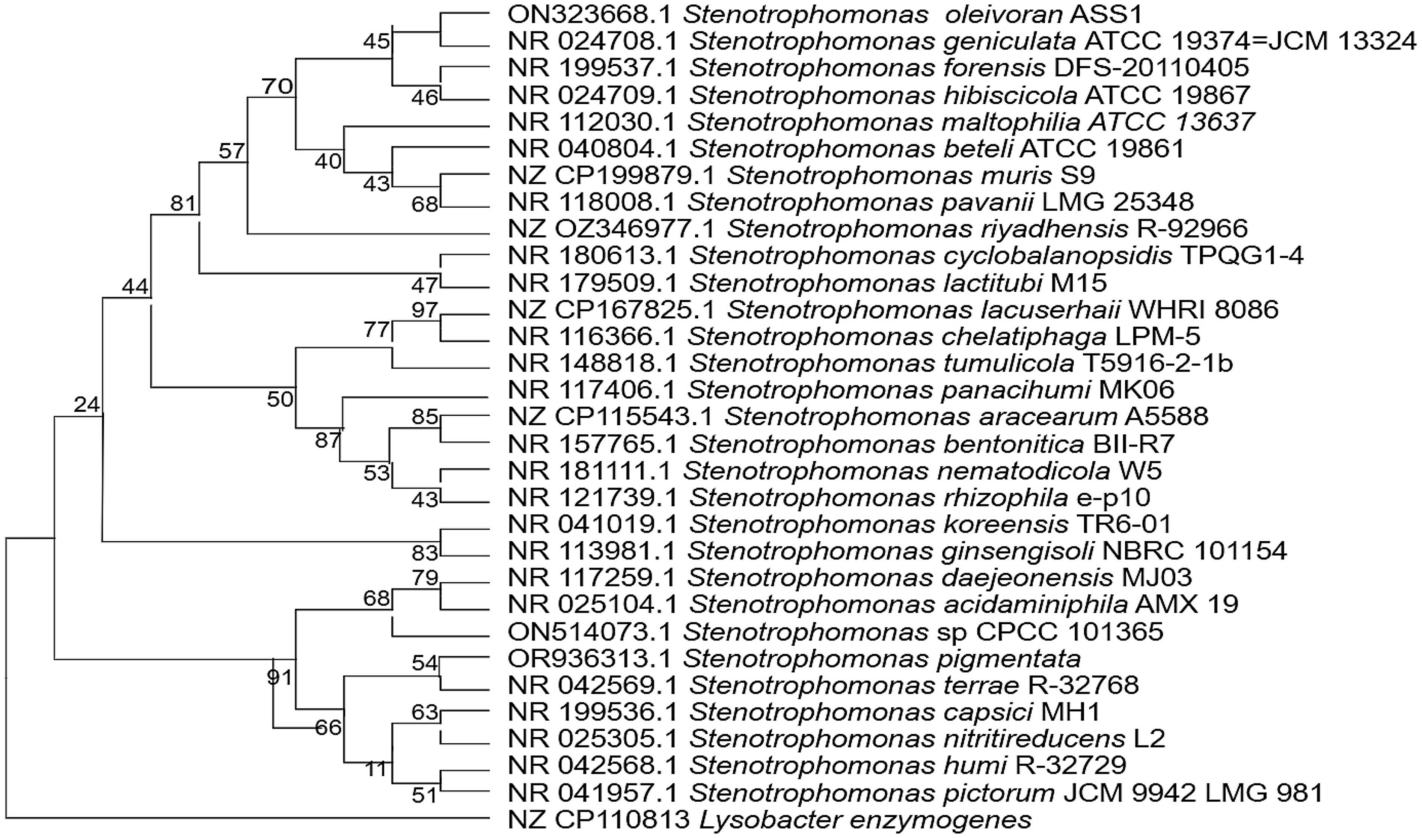
Maximum likelihood 16S rRNA gene phylogenetic tree showing the position of *Stenotrophomonas oleivorans* sp. nov. ASS1^T^ among the recognized *Stenotrophomonas* species. Constructed in MEGA XI using the Kimura 2-parameter model with 1,000 bootstrap replicates. Bootstrap values > = 50% are shown at the nodes. *Lysobacter enzymogenes* were used as the outgroup. Bar = 0.05 substitutions per nucleotide position.

The 16S rRNA gene (1,456 bp; GenBank: ON323668) shared 99.86% identity with *S. geniculata* ATCC 19374^T^ (= *Pseudomonas geniculata* ATCC 19374^T^ = JCM 13324^T^) and 74–99.86% identity across all *Stenotrophomonas* species. The ML tree (Figure 3) placed ASS1^T^ in a weakly supported clade with *S. geniculata* ATCC 19374^T^ (bootstrap 45%, below the ≥50% display threshold), together with *S. forensis* DFS-20110405 (bootstrap 70%) and *S. hibiscicola* ATCC 19867^T^ (bootstrap 46%). Most internal nodes had bootstrap support below 70%, reflecting the limited discriminatory power of the 16S rRNA marker at the species level within the genus *Stenotrophomonas*. Notably, *S. riyadhensis* R-92966, the closest relative to both ANIb (93.30%) and dDDH (54.3%), appeared in a distinct subclade and did not cluster with ASS1^T^, illustrating a 16S-genome discordance, a phenomenon that is well-documented in the *S. maltophilia* complex (Mignard and Flandrois, 2006; Janda and Abbott, 2007). High 16S rRNA identity does not indicate conspecificity, and genome-based analyses are required for reliable species delineation in this genus. autoMLST (Supplementary Figure S1 and Supplementary Table S2) and core-genome phylogeny (Supplementary Figure S2) both resolved ASS1^T^ as a distinct lineage from all type strains. Note: The clustering of ASS1^T^ with *S. geniculata* ATCC 19374^T^ (formerly *P. geniculata*) and *S. hibiscicola* ATCC 19867^T^ in the 16S ML tree reflects shared 16S sequence conservation and does not imply conspecificity; ASS1^T^ is oxidase-negative, consistent with *Stenotrophomonas* genus membership and with the reassigned *S. geniculata* ATCC 19374^T^ (Ryan et al., 2009).

### 3.5 Genome-based species delineation: ANI and dDDH

**Figure 4:**
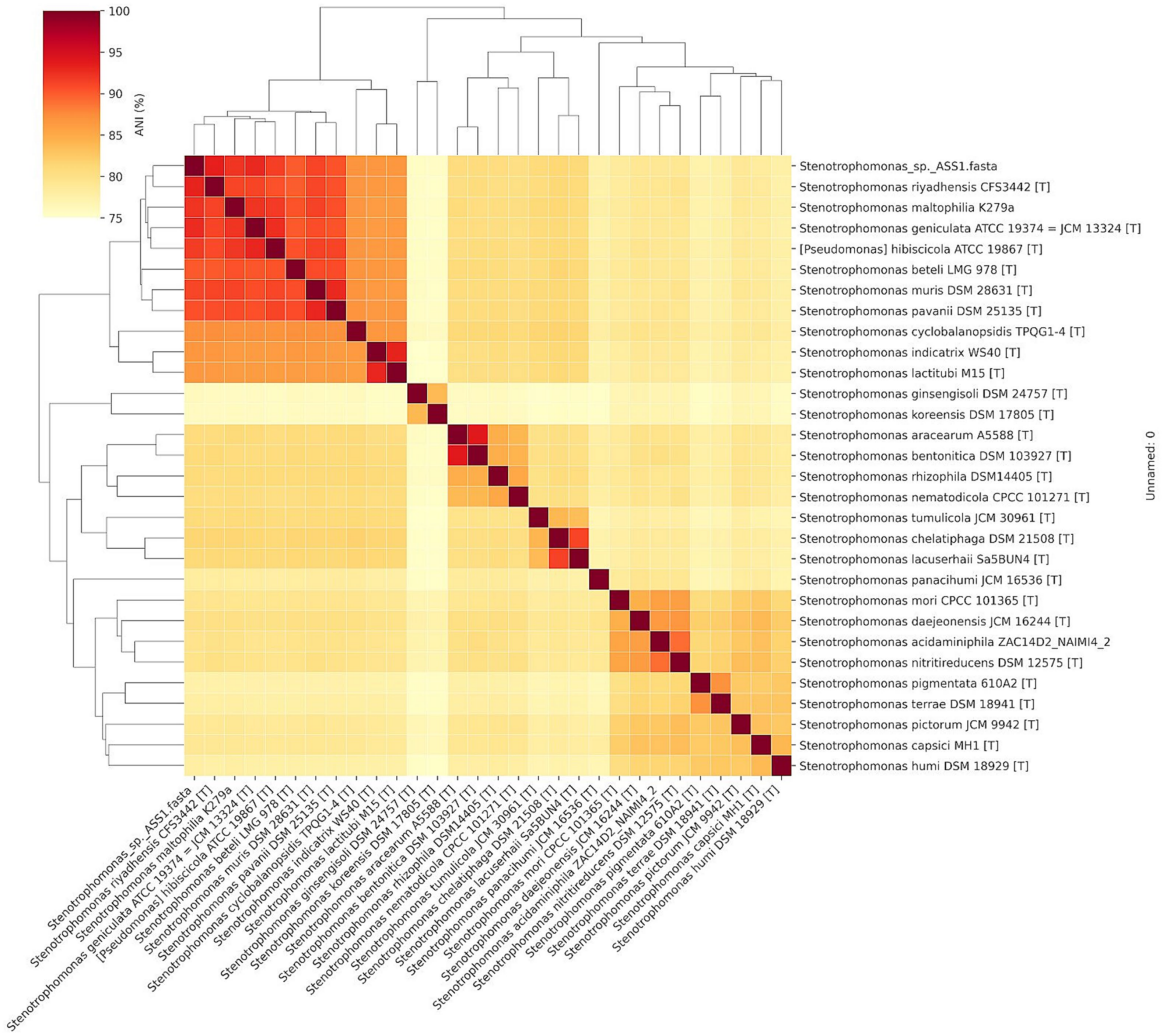
Heatmap of average nucleotide identity (ANI) values among ASS1T and 25 *Stenotrophomonas* type-strain genomes calculated using ANIb in JSpeciesWS. Color scale: ANI (%) from 75 (dark purple) to 100 (yellow); 95% species boundary is indicated. Hierarchical clustering by Ward linkage. ASS1T clusters closest to *S. geniculata* ATCC 19374 T (ANI 92.66%) but is clearly separated below the 95–96% species threshold.

Average nucleotide identity (ANI) analysis placed *S. riyadhensis* CFS3442^T^ as the closest relative by ANIb (93.30%), followed by *S. geniculata* ATCC 19374^T^ (92.74%), *S. maltophilia* K279a (92.16%), *S. hibiscicola* ATCC 19867^T^ (91.57%), *S. muris* DSM 28631^T^ (91.09%), and *S. pavanii* DSM 25135^T^ (90.47%). All ANI values were substantially below the 95–96% species threshold (Richter and Rosselló-Móra, 2009; Figure 4).

Digital DNA–DNA hybridization (dDDH) values between ASS1^T^ and 38 representative *Stenotrophomonas* and related genomes were calculated using GGDC v. 3.0 (Formula 2; Meier-Kolthoff et al., 2013), the recommended formula for genome-based species delineation (Table 2). The highest Formula 2 dDDH was 54.3% (95% CI: 51.6–57.0%; probability of DDH > =70%: 32.51%) against *Stenotrophomonas riyadhensis* ESTM1A, followed by *S. geniculata* ATCC 19374^T^ (= JCM 13324 T) at 50.4% (95% CI: 47.8–53.1%; probability of DDH > =70%: 19.53%), *S. maltophilia* ACYCa.6E at 48.1% (95% CI: 45.5–50.7%), *S. africana* ATCC 700475 at 47.7% (95% CI: 45.1–50.3%), and *P. hibiscicola* ATCC 19867 T at 46.0% (95% CI: 43.5–48.6%). All 38 Formula 2 values were well below the 70% species threshold, and the probability of DDH > =70% did not exceed 33% for any comparison, providing statistical confidence that ASS1^T^ is not conspecific with any recognized *Stenotrophomonas* species. TYGS independently classified ASS1T as a novel species. The full dDDH results for all 38 comparisons are presented in Table 2.

**Table 2:** Digital DNA–DNA hybridization (dDDH) values between *Stenotrophomonas oleivorans* sp. nov. ASS1ᵀ and 38 representative Stenotrophomonas and related species, estimated using the Genome-to-Genome Distance Calculator (GGDC v. 3.0; Meier-Kolthoff et al., 2013).

Reference genome	F1 dDDH (%)	F1 C. I.	F1 Prob. (≥70%)	F2 dDDH (%)	F2 C. I.	F2 Prob. (≥70%)	F3 dDDH (%)	F3 C. I.	F3 Prob. (≥70%)	ΔG+C (%)
*Stenotrophomonas riyadhensis ESTM1A MKCTX4 10a*	81.1	[77.2–84.4%]	92.0	**54.3**	**[51.6–57%]**	**32.5**	77.9	[74.4–81%]	93.2	0.12
***Stenotrophomonas geniculata ATCC 19374 T (= JCM 13324 T)***	77.2	[73.2–80.7%]	88.0	**50.4**	**[47.8–53.1%]**	**19.5**	73.4	[69.9–76.6%]	86.0	0.2
*Stenotrophomonas maltophilia ACYCa. 6E*	77.9	[74–81.5%]	88.7	**48.1**	**[45.5–50.7%]**	**14.2**	73.2	[69.7–76.4%]	84.4	0.07
*Stenotrophomonas maltophilia K279a*	75.8	[71.8–79.4%]	85.8	**48.0**	**[45.4–50.6%]**	**14.0**	71.4	[68–74.7%]	79.2	0.06
*Stenotrophomonas africana ATCC 700475*	78.9	[75–82.4%]	89.9	**47.7**	**[45.1–50.3%]**	**13.3**	73.9	[70.4–77.1%]	86.0	0.05
*Stenotrophomonas hibiscicola ATCC 19867*	77.3	[73.4–80.9%]	88.0	**46.0**	**[43.5–48.6%]**	**10.0**	71.9	[68.5–75.2%]	80.8	0.06
*Stenotrophomonas thermophila BurA1*	77.9	[73.9–81.4%]	88.7	**45.9**	**[43.4–48.5%]**	**9.8**	72.3	[68.9–75.6%]	82.0	0.18
*Stenotrophomonas muris S9*	75.2	[71.2–78.8%]	84.9	**44.8**	**[42.3–47.4%]**	**8.0**	69.8	[66.3–73%]	73.0	0.44
*Stenotrophomonas sepilia R-93097*	72.4	[68.4–76%]	79.6	**43.7**	**[41.2–46.3%]**	**6.4**	67.0	[63.6–70.3%]	60.6	0.24
*Stenotrophomonas pavanii YSM-1*	75.6	[71.6–79.2%]	85.5	**42.5**	**[39.9–45%]**	**4.8**	69.1	[65.6–72.3%]	70.1	1.00
*Stenotrophomonas forensis DFS-20110405*	70.7	[66.7–74.3%]	76.0	**42.4**	**[39.8–44.9%]**	**4.7**	65.1	[61.8–68.4%]	50.9	0.14
*Stenotrophomonas beteli LMG 978*	69.9	[66–73.6%]	74.1	**41.0**	**[38.5–43.5%]**	**3.4**	64.0	[60.6–67.2%]	44.6	0.46
*Stenotrophomonas tuberculopleuritidis 704A1*	65.1	[61.3–68.7%]	60.1	**34.7**	**[32.2–37.2%]**	**0.6**	57.4	[54.2–60.5%]	15.5	0.95
*Stenotrophomonas cyclobalanopsidis TPQG1-4*	57.4	[53.8–60.9%]	32.8	**34.0**	**[31.6–36.5%]**	**0.5**	51.4	[48.3–54.4%]	4.0	0.75
*Stenotrophomonas indicatrix DAIF1*	67.7	[63.8–71.3%]	68.1	**32.5**	**[30–35%]**	**0.3**	58.0	[54.8–61.2%]	17.8	0.02
*Stenotrophomonas lactitubi RW9*	71.1	[67.1–74.7%]	76.8	**32.4**	**[30–34.9%]**	**0.3**	60.4	[57.1–63.6%]	27.0	0.30
*Stenotrophomonas chelatiphaga*	37.0	[33.6–40.5%]	0.8	**25.4**	**[23.1–27.9%]**	**0.0**	33.1	[30.1–36.2%]	0.0	0.43
*Stenotrophomonas lacuserhaii Sa5BUN4*	36.7	[33.3–40.2%]	0.7	**25.2**	**[22.9–27.7%]**	**0.0**	32.8	[29.8–35.9%]	0.0	0.06
*Stenotrophomonas tumulicola JCM 30961*	33.5	[30.1–37%]	0.3	**25.2**	**[22.8–27.7%]**	**0.0**	30.4	[27.5–33.5%]	0.0	0.78
*Stenotrophomonas bentonitica DSM 103927*	33.6	[30.2–37.2%]	0.3	**25.0**	**[22.6–27.4%]**	**0.0**	30.5	[27.5–33.6%]	0.0	0.08
*Stenotrophomonas aracearum A5588*	34.3	[30.9–37.8%]	0.4	**24.8**	**[22.5–27.3%]**	**0.0**	30.9	[28–34%]	0.0	0.06
*Stenotrophomonas rhizophila DSM14405*	38.1	[34.8–41.6%]	1.1	**24.7**	**[22.3–27.1%]**	**0.0**	33.6	[30.7–36.7%]	0.0	0.92
*Stenotrophomonas oahuensis A5586*	31.6	[28.2–35.2%]	0.2	**24.4**	**[22.1–26.9%]**	**0.0**	28.8	[25.9–31.9%]	0.0	1.09
*Stenotrophomonas nematodicola CPCC 101271*	36.0	[32.6–39.5%]	0.6	**24.3**	**[22–26.7%]**	**0.0**	32.0	[29–35.1%]	0.0	0.95
*Stenotrophomonas daejeonensis JCM 16244*	26.9	[23.6–30.6%]	0.0	**23.8**	**[21.5–26.3%]**	**0.0**	25.2	[22.3–28.3%]	0.0	2.18
*Stenotrophomonas nitritireducens*	26.6	[23.2–30.2%]	0.0	**23.7**	**[21.4–26.2%]**	**0.0**	24.9	[22–28%]	0.0	2.11
*Stenotrophomonas acidaminiphila T0-18*	27.7	[24.4–31.4%]	0.0	**23.6**	**[21.3–26%]**	**0.0**	25.8	[22.9–28.9%]	0.0	2.79
*Stenotrophomonas capsici MH1*	27.2	[23.9–30.9%]	0.0	**23.3**	**[21.1–25.8%]**	**0.0**	25.3	[22.4–28.4%]	0.0	0.81
*Stenotrophomonas* sp. *ATCM1 4*	27.8	[24.4–31.4%]	0.0	**23.1**	**[20.9–25.6%]**	**0.0**	25.7	[22.8–28.8%]	0.0	0.77
*Stenotrophomonas mori CPCC 101365*	25.9	[22.5–29.5%]	0.0	**23.1**	**[20.8–25.6%]**	**0.0**	24.2	[21.4–27.3%]	0.0	3.79
*Stenotrophomonas pigmentata 610A2*	23.1	[19.8–26.7%]	0.0	**22.9**	**[20.6–25.3%]**	**0.0**	22.0	[19.2–25.1%]	0.0	3.09
*Stenotrophomonas terrae DSM 18941*	23.5	[20.2–27.1%]	0.0	**22.8**	**[20.5–25.2%]**	**0.0**	22.3	[19.5–25.4%]	0.0	2.50
*Stenotrophomonas pictorum JCM 9942*	25.6	[22.3–29.3%]	0.0	**22.6**	**[20.3–25.1%]**	**0.0**	23.9	[21.1–27%]	0.0	0.37
*Stenotrophomonas humi DSM 18929*	24.5	[21.2–28.1%]	0.0	**22.5**	**[20.2–24.9%]**	**0.0**	23.0	[20.2–26.1%]	0.0	2.34
*Stenotrophomonas panacihumi GSS15*	25.6	[22.2–29.2%]	0.0	**22.3**	**[20.1–24.8%]**	**0.0**	23.9	[21–27%]	0.0	2.40
*Stenotrophomonas ginsengisoli DSM 24757*	20.7	[17.5–24.3%]	0.0	**21.3**	**[19.1–23.7%]**	**0.0**	19.9	[17.2–23%]	0.0	0.50
*Stenotrophomonas koreensis DSM 17805*	20.3	[17.1–23.9%]	0.0	**21.0**	**[18.8–23.4%]**	**0.0**	19.5	[16.8–22.6%]	0.0	0.28
*Pseudoxanthomonas dokdonensis DSM 21858*	17.2	[14.2–20.8%]	0.0	**20.5**	**[18.3–23%]**	**0.0**	17.0	[14.4–20%]	0.0	1.90

The results were sorted in descending order using Formula 2 (d₄). Formula 2 is the recommended formula for whole-genome sequence-based species delineation. C. I. = 95% confidence interval; Prob. ≥70% = probability that DDH exceeds the 70% species threshold; ΔG + C = absolute G + C content difference (%). Green shading indicates strains with Formula 2 dDDH ≥40% (closest neighbors). No comparison reaches the 70% species boundary under Formula 2.

F1/F2/F3, GGDC Formulas 1, 2 (d₄), and 3. Green shading: Formula 2 dDDH ≥40% (closest neighbors). Bold blue italics: type strain used for ANI comparison. Red value: highest observed Formula 2 dDDH (54.3%). All Formula 2 values are below the 70% species delineation threshold.

Consistent with dDDH, JSpeciesWS ANIb analysis confirmed that *S. riyadhensis* CFS3442T was the closest relative (ANIb 93.30%), followed by *S. geniculata* ATCC 19374 T (92.74%). The convergence of both independent genomic metrics on the same nearest neighbor eliminates any method-dependent ambiguity and strengthens the novel species conclusion (Richter and Rosselló-Móra, 2009; Meier-Kolthoff et al., 2013). Both ANI and dDDH metrics independently confirm that ASS1^T^ is not conspecific with any recognized *Stenotrophomonas* species.

### 3.6 Pan-genome analysis

**Figure 5:**
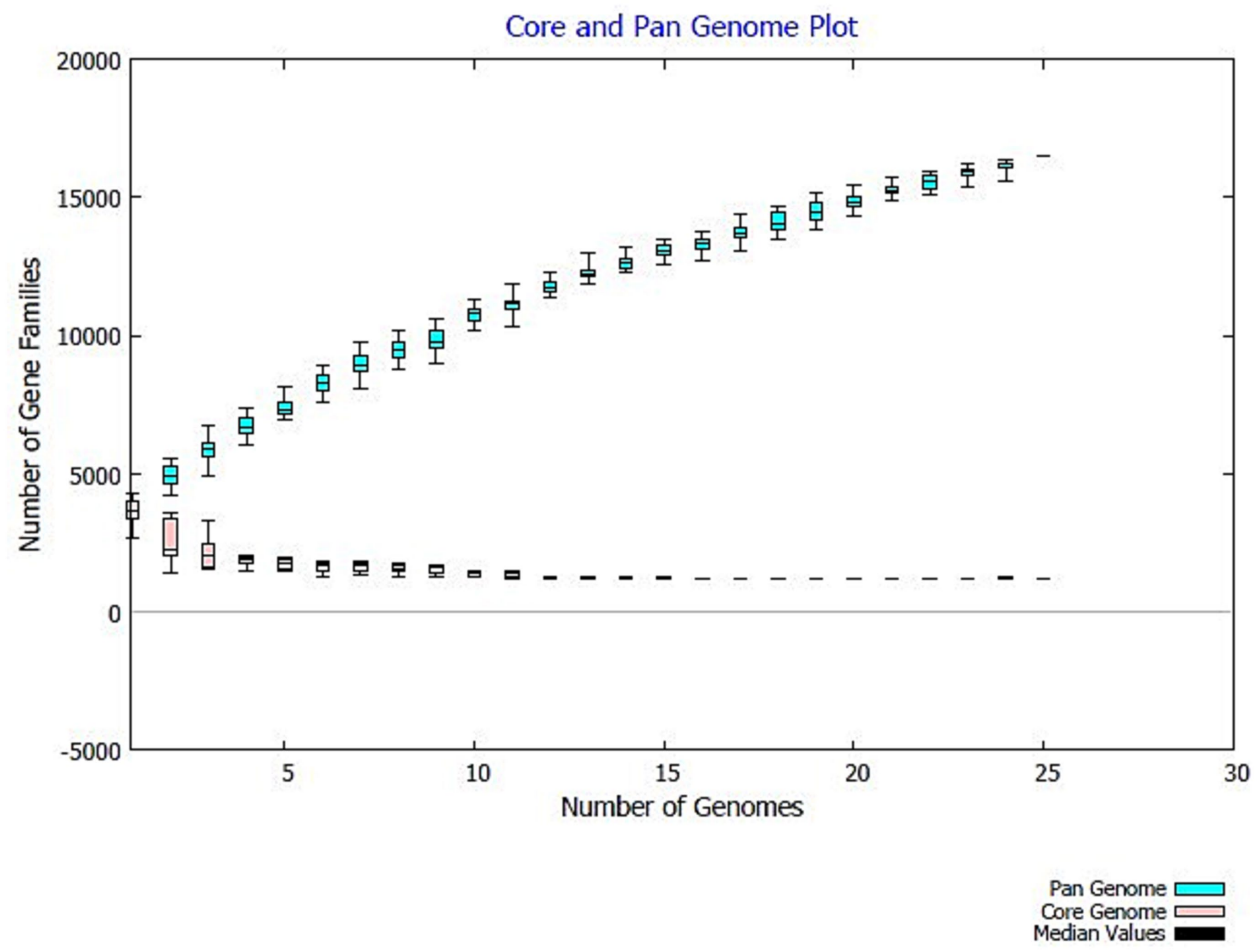
Core and pan-genome plots for ASS1^T^ and 24 representative *Stenotrophomonas genomes* generated using BPGA v. 1.3. The continuously rising pan-genome curve and alpha = 0.485 < 1 confirm an open pan-genome.

Pan-genome analysis of ASS1^T^ and 24 representative *Stenotrophomonas* genomes (BPGA v. 1.3) identified 1,177 core, 2,674 accessory, and 130 unique genes in ASS1^T^ (Figure 5). The pan-genome openness parameter alpha = 0.485 (< 1) confirms an open pan-genome, predicting the continued discovery of novel *Stenotrophomonas* species (Patil et al., 2018). Several unique genes in ASS1^T^ carry functional annotations relevant to environmental adaptation and hydrocarbon metabolism (Supplementary Tables S3–S5).

### 3.7 PAH degradation genes and *in vitro* growth on PAHs

Four PAH catabolic gene homologues were identified in the ASS1T genome: MG06_03320 (1,2-dihydroxy-1, 2-dihydronaphthalene dehydrogenase), MG068_17425 (2-hydroxychromene-2-carboxylate isomerase), MG_18055 (salicylaldehyde dehydrogenase), and MG068_20095 (naphthalene 1,2-dioxygenase). These genes encode enzymes involved in the upper pathway of aerobic naphthalene degradation via salicylate in the Tricarboxylic Acid (TCA) cycle (Das et al., 2015; Elufisan et al., 2020a, 2020b). KEGG pathway mapping revealed 44 hydrocarbon-metabolism genes in the core genome and 190 in the accessory genome (Supplementary Tables S3–S5).

Strain ASS1^T^ was isolated from crude oil-contaminated soil and demonstrated environmental persistence under field conditions. Under defined laboratory conditions, ASS1^T^ grew on anthracene, naphthalene, phenanthrene, and phenanthridine as the sole carbon sources in BH minimal medium. Descriptive statistics (22-day time series): anthracene, mean 9.85 × 10^7^ CFU/mL (SD 5.76 × 10^7); naphthalene, 7.75 × 10^7^ (SD 5.94 × 10^7^); phenanthrene, 1.16 × 10^8^ (SD 9.68 × 10^8^); phenanthridine, 8.82 × 10^7^ (SD 8.93 × 10^7^). The uninoculated PAH-only control showed no growth.

**Figure 6:**
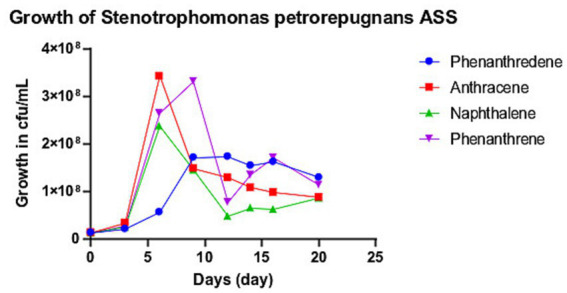
*In vitro* growth of *Stenotrophomonas oleivorans* sp. nov. ASS1T on four polycyclic aromatic hydrocarbons (PAHs) as sole carbon sources in Bushnell-Haas minimal medium (1 mg/mL; 30 °C; 200 rpm) over 22 days. Colony-forming units (CFU/mL) are determined every 2 days. The Kruskal–Wallis test detected no significant differences in growth among PAH substrates [H (3) = 2.403, *p* = 0.493].

Shapiro–Wilk tests revealed that normality was violated in three of the four PAH datasets (naphthalene W = 0.745, *p* = 0.002; phenanthrene W = 0.854, *p* = 0.041; phenanthridine W = 0.677, *p* < 0.001). Levene’s test confirmed equal variances [*F* (3, 44) = 0.569, *p* = 0.639]. The primary Kruskal–Wallis test detected no significant differences in growth among the four PAH substrates [H (3) = 2.403, *p* = 0.493]; this result was corroborated by one-way ANOVA [*F*(3, 44) = 0.536, *p* = 0.660]. All Mann–Whitney U pairwise comparisons were non-significant after Bonferroni correction (corrected alpha = 0.0083; all *p* > 0.21). Peak CFU/mL: phenanthridine 3.43 × 10^8 (day 2), phenanthrene 3.31 × 10^8 (day 4); anthracene supported the most sustained growth plateau (days 6–14; Figure 6).

## 4 Discussion

Polyphasic taxonomic analysis of strain ASS1^T^ demonstrated that it represents a novel *Stenotrophomonas* species. ANI of 93.30% (*vs. S. riyadhensis* CFS3442T; JSpeciesWS ANIb) and dDDH of 50.4% (GGDC Formula 2; vs. *S. geniculata* ATCC 19374 T; 95% CI: 47.8–53.1%) are unambiguously below the genomic species boundaries of > = 95–96% ANI and > = 70% dDDH (Richter and Rosselló-Móra, 2009). The highest Formula 2 dDDH observed in the complete 38-strain comparison was 54.3% against S. riyadhensis ESTM1A, a value that was well below the species threshold. The probability of dDDH > = 70% did not exceed 33% for any of the 38 comparisons, providing statistical confidence in the novel species assignment.

Although ASS1^T^ shares 99.86% 16S rRNA identity with *S. geniculata* ATCC 19374^T^ and groups near it in the 16S ML tree (bootstrap 45%, unsupported node), the genome-based metrics identify a different nearest neighbor. Both ANIb (93.30%) and dDDH (54.3%) independently identified *S. riyadhensis* CFS3442^T^ as the closest valid published relative; it occupies a distinct subclade in the 16S tree. This discordance between 16S rRNA topology and genome-based metrics is a well-documented phenomenon within the *Stenotrophomonas maltophilia* complex, where the 16S marker lacks sufficient resolution to reflect true genomic relationships, and most interspecies nodes have bootstrap support below 70% (Janda and Abbott, 2007; Patil et al., 2018; Mignard and Flandrois, 2006). The convergence of two independent genome-based methods on the same nearest neighbor (*S. riyadhensis*), combined with low 16S bootstrap support, strongly supports prioritizing genome-based species delineation in this genus. Both GGDC Formula 2 dDDH (54.3%) and JSpeciesWS ANIb (93.30%) independently identified *S. riyadhensis* CFS3442T as the closest validly published relative, with *S. geniculata* ATCC 19374 T ranking second by both metrics (ANIb 92.74%; dDDH 50.4%). The convergence of these independent genomic approaches to the same nearest neighbor strengthens the novel species conclusion (Richter and Rosselló-Móra, 2009; Meier-Kolthoff et al., 2013).

Biochemically, ASS1^T^ is distinguished from *S. geniculata* ATCC 19374^T^ and *S. hibiscicola* ATCC 19867^T^ by its inability to hydrolyze gelatin and its ability to utilize galactose. The fatty acid profiles differed substantially: iso-C16:0, iso-C17:1w9c, C18:1w9c, and C19 are present in ASS1^T^ but absent in both reference strains, whereas anteiso-C15:0, C15:0, iso-C13:0 3-OH, and C13:0 2-OH are absent in ASS1^T^ but present in the reference strains. These chemotaxonomic differences provide independent phenotypic support for genomic separation.

The predominant fatty acids in ASS1^T,^ C16:0, iso-C12:0, iso-C16:0, iso-C17:1w9c, C18:0, C18:1w9c (oleic acid), and C19, carry functional significance beyond their chemotaxonomic utility. The high abundance of C16:0 (palmitic acid) is a hallmark of Gammaproteobacteria and is consistent with *Stenotrophomonas* fatty acid profiles reported by Ryan et al. (2009) and Patil et al. (2018). The branched-chain fatty acids iso-C12:0 and iso-C16:0 are indicators of cold-adaptive and membrane fluidity regulatory mechanisms. Bacteria increase the proportion of branched-chain and unsaturated fatty acids in the inner membrane in response to environmental stress, including exposure to organic solvents and aromatic hydrocarbons, to maintain membrane integrity and fluidity under toxic conditions (Segura et al., 2017; Ramos et al., 2011). The detection of iso-C17:1w9c (an unusually branched monounsaturated fatty acid) and C18:1w9c (oleic acid) in ASS1^T^ is particularly relevant. Oleic acid and its isomers are associated with solvent-tolerant phenotypes in environmental bacteria, and their presence in the ASS1^T^ membrane may contribute to its capacity to survive and grow in crude oil-contaminated environments, where PAH concentrations can reach phytotoxic levels (Segura et al., 2017). C19, detected as a minor but reproducible component, is consistent with a cyclopropane fatty acid (C19:0 cyclopropane), which Gram-negative bacteria synthesize from C18:1 precursors during the stationary phase and under stress conditions as a membrane rigidification response; elevated cyclopropane fatty acid levels have been reported in PAH-exposed bacteria and are thought to protect against stress-induced membrane perturbation (Pini et al., 2009). In summary, the fatty acid profile of ASS1^T^ is consistent with a membrane composition adapted for persistence in hydrocarbon-rich environments, complementing the genomic evidence of PAH catabolic capacity and further supporting the ecological origin of the strain from crude oil-contaminated soil.

The ASS1^T^ genome encodes a complete upper-pathway cassette for aerobic PAH catabolism via the naphthalene pathway: naphthalene 1,2-dioxygenase (MG068_20095), which catalyzes the initial deoxygenation of the aromatic ring; 1,2-dihydroxy-1,2-dihydronaphthalene dehydrogenase (MG06_03320), which oxidizes the cis-diol intermediate; 2-hydroxychromene-2-carboxylate isomerase (MG068_17425), a rearrangement enzyme in the lower salicylate branch; and salicylaldehyde dehydrogenase (MG_18055), which converts salicylaldehyde to salicylate for entry into the TCA cycle (Das et al., 2015; Elufisan et al., 2020b). The presence of this complete four-enzyme cassette is noteworthy because incomplete upper-pathway gene sets are common in environmental isolates and typically result in dead-end metabolite accumulation rather than productive catabolism (Pal et al., 2017). The naphthalene 1,2-dioxygenase homologues of ASS1^T^ share sequence conservation with those characterized in Stenotrophomonas sp. Pemsol, another crude oil-associated Stenotrophomonas isolate carrying the same four-gene cassette (Elufisan et al., 2020b), suggesting that this gene configuration may be conserved across oil-contaminated soil *Stenotrophomonas* ecotypes. KEGG pathway mapping further identified 44 hydrocarbon-metabolism genes in the core genome and 190 in the accessory genome (Supplementary Tables S3–S5), indicating that PAH catabolism is one component of a broader hydrocarbon metabolism repertoire in ASS1^T^.

*In vitro* growth assays confirmed that ASS1^T^ uses anthracene, naphthalene, phenanthrene, and phenanthridine as the sole carbon sources in Bushnell-Haas minimal medium. Peak cell densities were achieved on phenanthridine (3.43 × 10^8^ CFU/mL at day 2) and phenanthrene (3.31 × 10^8 CFU/mL at day 4), both three-ring PAHs, while naphthalene (a two-ring compound) supported a lower peak density. Anthracene sustained the most prolonged growth plateau (days 6–14), consistent with its lower aqueous solubility and slower bioavailability compared to naphthalene (Das et al., 2015). Kruskal–Wallis analysis detected no statistically significant difference in growth across the four substrates [H (3) = 2.403, *p* = 0.493]. This result indicates that ASS1^T^ employs a substrate-generalist *in vitro* catabolic strategy, in contrast to the substrate-specialist preference observed in dedicated PAH degraders, such as *Sphingomonas and Mycobacterium* spp. (Pal et al., 2017). This broad-spectrum in vitro catabolic capacity compares favorably with published data for other *Stenotrophomonas* PAH degraders. *Stenotrophomonas* sp. Pemsol, the most closely characterized oil-soil *Stenotrophomonas* isolate, degraded naphthalene and phenanthrene but was not evaluated against anthracene or phenanthridine (Elufisan et al., 2020b). *Stenotrophomonas maltophilia* strains have been documented to degrade naphthalene and fluorene under similar minimal medium conditions but typically show markedly reduced growth on three-ring PAHs relative to two-ring substrates, whereas ASS1^T^ exhibited statistically equivalent growth across both ring-size classes (Geng et al., 2010; Patel, 2016). Growth on phenanthridine is particularly uncommon among *Stenotrophomonas* species. This nitrogen-containing three-ring polycyclic compound has not been reported as a growth substrate for *S. maltophilia* or Pemsol, making ASS1^T^ one of the few members of the genus to utilize phenanthridine as a sole carbon source under laboratory conditions.

These findings should be contextualized within the constraints of in vitro experimentation. Growth in BH minimal medium supplemented with individual pure PAHs does not replicate the physicochemical and biological complexity of crude oil-contaminated soil, which contains multi-component hydrocarbon mixtures, competing and synergistic microbial communities, surfactant-mediated bioavailability gradients, and nutrient regimes that are absent from the BH medium. The environmental persistence of ASS1^T^ is established by its isolation from contaminated soils. *In vitro* growth on PAHs and the presence of a complete catabolic gene cassette constitute molecular and physiological evidence of the degradation potential under defined conditions. Validation of *in situ* degradation activity would require mesocosm or field experiments employing isotope-labelled PAH tracers or metatranscriptomic confirmation of catabolic gene expression (Das et al., 2015). Within these constraints, the combination of a broad in vitro substrate range spanning two- and three-ring PAHs (including the recalcitrant nitrogen-containing compound phenanthridine), a complete four-enzyme upper catabolic pathway, an open pan-genome with 190 accessory hydrocarbon metabolism genes, and isolation from crude oil-contaminated soil collectively support ASS1^T^ as a candidate organism for future bioremediation research targeting multi-PAH-contaminated environments.

## 5 Taxonomic conclusion and description of *Stenotrophomonas oleivorans* sp. nov.

### 5.1 Taxonomic conclusion

Based on phylogenetic, genomic (ANI and dDDH), phenotypic, and chemotaxonomic evidence, *Stenotrophomonas* sp. ASS1^T^ represents a novel *Stenotrophomona*s species. We formally propose *Stenotrophomonas oleivorans* sp. nov., with strain ASS1T as the type strain.

### 5.2 Description of *Stenotrophomonas oleivorans* sp. nov.

*Stenotrophomonas oleivorans* (o.le.i.vo’rans. L. neut. n. oleum, oil; L. part. Adj. vorans, from vorare, to devour; N. L. part. Adj. *oleivorans*), oil-devouring, referring to the capacity to utilize petroleum-derived polycyclic aromatic hydrocarbons as sole carbon sources in a defined laboratory medium.

Gram-negative, motile, non-spore-forming rods (0.5–0.8 × 1.5–3.0 um). Colonies on LB and SVIA agar are yellow, slightly raised, smooth, circular after 48 h at 37 °C, and colorless on MacConkey agar (non-lactose fermenter). Growth at 25–37 °C (optimum 37 °C) and pH 6–8 (optimum pH 7–8). No growth at 42 °C.

Catalase-positive; oxidase-negative. Hydrolyzes starch, Tween 80, and aesculin; does not hydrolyze gelatin. Utilizes arabinose, glucose, fructose, mannose, galactose, lysine, and citrate as the sole carbon sources. Non-lactose fermenter. Resistant to sulfamethoxazole-trimethoprim, chloramphenicol, imipenem, ceftriaxone, ceftazidime, ampicillin, doxycycline, gentamicin, and nitrofurantoin; susceptible to levofloxacin and ofloxacin.

The dominant fatty acids were C16:0, iso-C12:0, iso-C16:0, iso-C17:1w9c, C18:0, C18:1w9c, and C19 (Supplementary Table S1). Complete genome: 4,564,481 bp, G + C content: 66.59%, single closed contig (GenBank: CP031167; N50 = 4,564,481 bp). BUSCO completeness 99.7% (gammaproteobacteria_odb10; 365/366 BUSCOs complete), CheckM2 completeness 100.0%, and contamination 0.0%. Encodes 4,104 CDSs (coding density 0.893), genes for enzymes of PAH catabolism (naphthalene 1,2-dioxygenase, 1,2-dihydroxy-1,2-dihydronaphthalene dehydrogenase, 2-hydroxychromene-2-carboxylate isomerase, salicylaldehyde dehydrogenase), and six BGCs, including one NRPS cluster. ANI with the closest type strain *S. riyadhensis* CFS3442T: 93.30% (JSpeciesWS ANIb); *S. geniculata* ATCC 19374 T (= JCM 13324 T): 92.74%; dDDH (GGDC Formula 2): 50.4% [95% CI: 47.8–53.1%], both below species delineation thresholds.

The strain grows on naphthalene, phenanthrene, anthracene, biphenyl, anthraquinone, and phenanthridine as sole carbon sources in the BH minimal medium under laboratory conditions.

Type strain ASS1^T^ (= WFCC 1006/CM-CNRG 934 T) was isolated from crude oil-contaminated soil in Villa Hermosa, Tabasco, Mexico (17° 59′13” N, 92° 55′10” W). 16S rRNA gene sequence: GenBank accession number ON323668.

## Data Availability

The data presented in this study are publicly available in the NCBI (https://www.ncbi.nlm.nih.gov/), accession CP031167.

## Footnotes

1
https://lpsn.dsmz.de/genus/stenotrophomonas

2
https://automlst.ziemertlab.com

## References

[ref1] Aladame N. (1987). Bergey’s manual of systematic bacteriology. Ann. Inst. Pasteur Microbiol. 138:146. doi: 10.1016/0769-2609(87)90099-8

[ref2] Auch A. F. von Jan M. Klenk H. P. Göker M. (2010). Digital DNA-DNA hybridization for microbial species delineation by means of genome-to-genome sequence comparison. Stand. Genomic Sci. 2, 117–134. doi: 10.4056/sigs.531120, 21304684 PMC3035253

[ref3] Bankevich A. Nurk S. Antipov D. Gurevich A. A. Dvorkin M. Kulikov A. S. . (2012). SPAdes: a new genome assembly algorithm and its applications to single-cell sequencing. J. Comput. Biol. 19, 455–477. doi: 10.1089/cmb.2012.0021, 22506599 PMC3342519

[ref4] Blin K. Shaw S. Kloosterman A. M. Charlop-Powers Z. Van Wezel G. P. Medema M. H. . (2021). AntiSMASH 6.0: improving cluster detection and comparison capabilities. Nucleic Acids Res. 49, W29–W35. doi: 10.1093/nar/gkab335, 33978755 PMC8262755

[ref5] Breed Robert S Murray E.G.D Smith Nathan R (1957). Bergeys-manual-of-Determinativ-bacteriology-seventh-edition. xviii+–1094.

[ref6] Brian B. L. Gardner E. W. (1967). A simple procedure for detecting the presence of cyclopropane fatty acids in bacterial lipids. Applied microbiology, 16, 549–552., 4869615 10.1128/am.16.4.549-552.1968 PMC547466

[ref7] Chaudhari N. M. Gupta V. K. Dutta C. (2016). BPGA-an ultra-fast pan-genome analysis pipeline. Sci. Rep. 6:24373. doi: 10.1038/srep24373, 27071527 PMC4829868

[ref8] Chklovski A. Parks D. H. Woodcroft B. J. Tyson G. W. (2023). CheckM2: a rapid, scalable and accurate tool for assessing microbial genome quality using machine learning. Nat. Methods 20, 1203–1212. doi: 10.1038/s41592-023-01940-w 37500759

[ref9] Coenye T. Vanlaere E. Falsen E. Vandamme P. (2004). *Stenotrophomonas africana* Drancourt 1997 is a later synonym of *Stenotrophomonas maltophilia* (Hugh 1981) Palleroni and Bradbury 1993. Int. J. Syst. Evol. Microbiol. 54, 1235–1237. doi: 10.1099/ijs.0.63093-0 15280297

[ref10] Das D. Baruah R. Sarma Roy A. Singh A. K. Deka Boruah H. P. Kalita J. . (2015). Complete genome sequence analysis of *Pseudomonas aeruginosa* N002 reveals its genetic adaptation for crude oil degradation. Genomics 105, 182–190. doi: 10.1016/j.ygeno.2014.12.006, 25546474

[ref11] Denton M. Kerr K. G. (1998). Microbiological and clinical aspects of infection associated with. Microbiology 11, 57–80. 10.1128/cmr.11.1.57 PMC121376 9457429

[ref12] Elbir H. (2024). Updating the relationship between the threshold value of average nucleotide identity and digital DNA–DNA hybridization for reliable taxonomy of Corynebacterium. Vet. Sci. 11:661. doi: 10.3390/vetsci11120661 39729001 PMC11680202

[ref13] Elufisan T. O. Lozano L. Bustos P. Rodríguez-Luna I. C. Sánchez-Varela A. Oyedara O. O. . (2019). Complete genome sequence of *Stenotrophomonas maltophilia* strain SVIA2, isolated from crude oil-contaminated soil in Tabasco, Mexico. Microbiol. Resour. Announc 8:e00529–19. doi: 10.1128/mra.00529-19 31346014 PMC6658684

[ref14] Elufisan T. O. Luna I. C. R. Oyedara O. O. Varela A. S. García V. B. Oluyide B. O. . (2020a). Antimicrobial susceptibility pattern of stenotrophomonas species isolated from Mexico. Afr. Health Sci. 20, 168–181. doi: 10.4314/ahs.v20i1.22 33402905 PMC7750080

[ref15] Elufisan T. O. Rodríguez-Luna I. C. Oyedara O. O. Sánchez-Varela A. Hernández-Mendoza A. Dantán Gonzalez E. . (2020b). The polycyclic aromatic hydrocarbon (PAH) degradation activities and genome analysis of a novel strain Stenotrophomonas sp. Pemsol isolated from Mexico. PeerJ 8:e8102. doi: 10.7717/peerj.8102, 31934497 PMC6951288

[ref16] Gautam V. Patil P. P. Bansal K. Kumar S. Kaur A. Singh A. . (2021a). Description of Stenotrophomonas sepilia sp. nov., isolated from blood culture of a hospitalized patient as a new member of *Stenotrophomonas maltophilia* complex. New Microb. New Infect. 43:100920. doi: 10.1016/j.nmni.2021.100920, 34457314 PMC8379335

[ref17] Gautam V. Patil P. P. Bansal K. Kumar S. Kaur A. Singh A. . (2021b). Description of *Stenotrophomonas sepilia* sp. nov., isolated from blood culture of a hospitalized patient as a new member of *Stenotrophomonas maltophilia* complex. New Microb. New Infect. 43:100920. doi: 10.1016/j.nmni.2021.100920, 34457314 PMC8379335

[ref18] Geng Y. Wang K. Chen D. Huang X. He M. Yin Z. (2010). *Stenotrophomonas maltophilia*, an emerging opportunist pathogen for cultured channel catfish, *Ictalurus punctatus*, in China. Aquaculture 308, 132–135. doi: 10.1016/j.aquaculture.2010.08.032

[ref19] Grant J. R. Enns E. Marinier E. Mandal A. Herman E. K. Chen C. Y. . (2023). Proksee: in-depth characterization and visualization of bacterial genomes. Nucleic Acids Res. 51, W484–W492. doi: 10.1093/nar/gkad326, 37140037 PMC10320063

[ref20] Hugh R. Leifson E. (1963). A description of the type strain of *Pseudomonas maltophilia*. Int. Bull. Bacteriol. Nomencl. Taxon. 13, 133–138. doi: 10.1099/0096266X-13-3-133

[ref21] Hugh R. Ryschenkow E. (1961). *Pseudomonas maltophilia*, an Alcaligenes-like species. J. Gen. Microbiol. 26, 123–132. doi: 10.1099/00221287-26-1-123 14449786

[ref22] Janda J. M. Abbott S. L. (2007). 16S rRNA gene sequencing for bacterial identification in the diagnostic laboratory: pluses, perils, and pitfalls. J. Clin. Microbiol. 45, 2761–2764. doi: 10.1128/JCM.01228-07, 17626177 PMC2045242

[ref23] Komagata K. Yabuuchi E. Tamagawa Y. Ohyama A. (1974). Pseudomonas melanogena Iizuka and Komagata 1963, a later subjective synonym of *Pseudomonas maltophilia* Hugh and Ryschenkow 1960. Int. J. Syst. Bacteriol. 24, 242–247. doi: 10.1099/00207713-24-2-242

[ref24] Lee I. Chalita M. Ha S. M. Na S. I. Yoon S. H. Chun J. (2017). ContEst16S: an algorithm that identifies contaminated prokaryotic genomes using 16S RNA gene sequences. Int. J. Syst. Evol. Microbiol. 67, 2053–2057. doi: 10.1099/ijsem.0.001872, 28639931

[ref25] Lee D. S. Ryu S. H. Hwang H. W. Kim Y. J. Park M. Lee J. R. . (2008). *Pseudoxanthomonas sacheonensis* sp. nov., isolated from BTEX-contaminated soil in Korea, transfer of *Stenotrophomonas dokdonensis* yoon 2006 to the genus Pseudoxanthomonas as *Pseudoxanthomonas dokdonensis* comb. nov. and emended description of the genes. Int. J. Syst. Evol. Microbiol. 58, 2235–2240. doi: 10.1099/ijs.0.65678-0 18768635

[ref26] Mangwani N. Shukla S. K. Kumari S. Rao T. S. Das S. (2014). Characterization of *Stenotrophomonas acidaminiphila* NCW-702 biofilm for implication in the degradation of polycyclic aromatic hydrocarbons. J. Appl. Microbiol. 117, 1012–1024. doi: 10.1111/jam.12602, 25040365

[ref27] Manni M. Berkeley M. R. Seppey M. Zdobnov E. M. (2021). BUSCO: assessing genomic data quality and beyond. Curr. Protoc. 1:e323. doi: 10.1002/cpz1.323, 34936221

[ref9002] Mavromatis K. Ivanova N. N. Chen I. M. A. Szeto E. Markowitz V. M. Kyrpides N. C. (2009). The DOE-JGI Standard operating procedure for the annotations of microbial genomes. Standards in genomic sciences, 1, 63–67. 21304638 10.4056/sigs.632 PMC3035208

[ref28] Meier-Kolthoff J. P. Auch A. F. Klenk H. P. Göker M. (2013). Genome sequence-based species delimitation with confidence intervals and improved distance functions. BMC Bioinform. 14:60. doi: 10.1186/1471-2105-14-60, 23432962 PMC3665452

[ref29] Meier-Kolthoff J. P. Göker M. (2019). TYGS is an automated high-throughput platform for state-of-the-art genome-based taxonomy. Nat. Commun. 10, 2182–2110. doi: 10.1038/s41467-019-10210-3, 31097708 PMC6522516

[ref30] Mignard S. Flandrois J. P. (2006). 16S rRNA sequencing in routine bacterial identification: a 30-month experiment. J. Microbiol. Methods 67, 574–581. doi: 10.1016/j.mimet.2006.05.009 16859787

[ref31] Nesme X. Vaneechoutte M. Orso S. Hoste B. Swings J. (1995). Diversity and genetic relatedness within genera *Xanthomonas* and *Stenotrophomonas* using restriction endonuclease site differences of PCR-amplified 16S rRNA gene. Syst. Appl. Microbiol. 18, 127–135. doi: 10.1016/S0723-2020(11)80460-1

[ref32] Oyedara O. O. De Luna-Santillana E. d. J. Olguin-Rodriguez O. Guo X. Mendoza-Villa M. A. Menchaca-Arredondo J. L. . (2016). Isolation of *Bdellovibrio* sp. from soil samples in Mexico and their potential applications in control of pathogens. Microbiology 5, 992–1002. doi: 10.1002/mbo3.382, 27297185 PMC5221441

[ref33] Pal S. Kundu A. Banerjee T. D. Mohapatra B. Roy A. Manna R. . (2017). Genome analysis of crude oil degrading *Franconibacter pulveris* strain DJ34 revealed its genetic basis for hydrocarbon degradation and survival in oil contaminated environment. Genomics 109, 374–382. doi: 10.1016/j.ygeno.2017.06.002, 28625866

[ref9001] Palleroni N. J. Bradbury J. F. (1993). Stenotrophomonas, a new bacterial genus for Xanthomonas maltophilia (Hugh 1980) Swings et al. 1983, *International journal of systematic and evolutionary microbiology*, 43, 606–609. 10.1099/00207713-43-3-606 8347518

[ref34] Palleroni N. J. Kunisawa R. Contopoulou R. Doudoroff M. (1973). Nucleic acid homologies in the genus *Pseudomonas*. Int. J. Syst. Bacteriol. 23, 333–339. doi: 10.1099/00207713-23-4-333

[ref35] Patel J. B. (2016). Performance Standards for Antimicrobial Susceptibility Testing. Malvern, PA: Clinical and Laboratory Standards Institute.

[ref36] Patil P. P. Kumar S. Midha S. Gautam V. Patil P. B. (2018). Taxonogenomics reveal multiple novel genomospecies associated with clinical isolates of *Stenotrophomonas maltophilia*. Microb. Genom. 4:e000207. doi: 10.1099/mgen.0.000207, 30084764 PMC6159553

[ref37] Pini C. V. Bernal P. Godoy P. Ramos J. L. Segura A. (2009). Cyclopropane fatty acids are involved in organic solvent tolerance but not in acid stress resistance in *Pseudomonas putida* DOT-T1E. Microb. Biotechnol. 2, 253–261. doi: 10.1111/j.1751-7915.2009.00084.x, 21261919 PMC3815845

[ref38] Ramos P. L. Van Trappen S. Thompson F. L. Rocha R. C. S. Barbosa H. R. de Vos P. . (2011). Screening for endophytic nitrogen-fixing bacteria in Brazilian sugar cane varieties used in organic farming and description of *stenotrophomonas Pavanii* sp. nov. Int. J. Syst. Evol. Microbiol. 61, 926–931. doi: 10.1099/ijs.0.019372-0, 20495025

[ref39] Řezanka T. Kambourova M. Derekova A. Kolouchová I. Sigler K. (2012). LC-ESI-MS/MS identification of polar lipids of two thermophilic Anoxybacillus bacteria containing a unique lipid pattern. Lipids 47, 729–739. doi: 10.1007/s11745-012-3675-0, 22566206

[ref40] Richter M. Rosselló-Móra R. (2009). Shifting the genomic gold standard for the prokaryotic species definition. Proc. Natl. Acad. Sci. 106, 19126–19131. doi: 10.1073/PNAS.0906412106, 19855009 PMC2776425

[ref41] Richter M. Rosselló-Móra R. Oliver Glöckner F. Peplies J. (2016). JSpeciesWS: a web server for prokaryotic species circumscription based on pairwise genome comparison. Bioinformatics 32, 929–931. doi: 10.1093/bioinformatics/btv681, 26576653 PMC5939971

[ref42] Ryan R. P. Monchy S. Cardinale M. Taghavi S. Crossman L. Avison M. B. . (2009). The versatility and adaptation of bacteria from the genus Stenotrophomonas. Nat. Rev. Microbiol. 7, 514–525. doi: 10.1038/nrmicro2163, 19528958

[ref43] Sánchez-Castro I. Bakkali M. Merroun M. L. (2017). Draft genome sequence of *Stenotrophomonas bentonitica* BII-R7 T, a selenite-reducing bacterium isolated from Spanish bentonites. Genome Announc. 5:e00719–17. doi: 10.1128/genomea.00719-17, 28774976 PMC5543638

[ref44] Seemann T. (2014). Prokka: rapid prokaryotic genome annotation. Bioinformatics 30, 2068–2069. doi: 10.1093/bioinformatics/btu153, 24642063

[ref45] Segura A. Hernández-Sánchez V. Marqués S. Molina L. (2017). Insights in the regulation of the degradation of PAHs in Novosphingobium sp. HR1a and utilization of this regulatory system as a tool for the detection of PAHs. Sci. Total Environ. 590–591, 381–393. doi: 10.1016/j.scitotenv.2017.02.180, 28285855

[ref46] Swings J. De Vos P. den Van Mooter M. De Ley J. (2009). Transfer of *Pseudomonas maltophilia* Hugh 1981 to the genus *Xanthomonas* as *Xanthomonas maltophilia* (Hugh 1981) comb. nov. Int. J. Syst. Bacteriol. 33, 409–413. doi: 10.1099/00207713-33-2-409

[ref47] Tatusova T. Dicuccio M. Badretdin A. Chetvernin V. Nawrocki E. P. Zaslavsky L. . (2016). NCBI prokaryotic genome annotation pipeline. Nucleic Acids Res. 44, 6614–6624. doi: 10.1093/nar/gkw569, 27342282 PMC5001611

[ref48] Ulrich J. A. Needham G. M. (1953). Differentiation of *Alcaligenes faecalis* from *Brucella bronchisepticus* by biochemical and nutritional methods. J. Bacteriol. 65, 210–215. doi: 10.1128/jb.65.2.210-215.1953, 13034718 PMC169668

[ref49] Yang P. De Vos P. Kersters K. Swings J. (1993). Polyamine patterns as chemotaxonomic markers for the genus *Xanthomonas*. Int. J. Syst. Bacteriol. 43, 709–714.

